# The Political Economy of Circular Economies: Lessons from Future Repair Scenario Deliberations in Sweden

**DOI:** 10.1007/s43615-021-00128-8

**Published:** 2021-11-10

**Authors:** Johan Niskanen, Duncan McLaren

**Affiliations:** 1 Department of Thematic Studies – Technology and Social Change, Linköping University, 581 83 Linköping, Sweden; 2 Lancaster Environment Centre, Lancaster University, Library Avenue, Lancaster, LA1 4YQ UK

**Keywords:** Circular economy, Sociology of repair, Political economy; Scenario workshop

## Abstract

The dominant technocratic and neoliberal imaginary of a circular economy dependent on corporate leadership, market mechanisms, and changed consumer behaviour is here explored using the findings of deliberative stakeholder workshops examining diverse scenarios for the promotion of repair as part of a circular economy. Stakeholder responses to four scenarios—digital circularity, planned circularity, circular modernism, and bottom-up sufficiency—are described with reference to the ideologies, interests, and institutions involved. We distinguish two levels of discourse in the stakeholder discussions. The main narrative in which individualist and consumerist ideologies dominate, even within ideals of sustainability, reflects a conjunction of corporate, labour, and public interests in the market liberal social democratic state, with proposed interventions focused on the institutions of markets and education. A subaltern narrative present in the margins of the discussions challenges the consumerist and productivist presumptions of the market liberal political economy and hints at more transformative change. These conflicting responses not only cast light on the ways in which the political economy of contemporary Sweden (within the European Union) constrains and conditions current expectations and imaginaries of circularity, but also suggest ways in which the future political economy of circular economies might be contested and evolve.

**Supplementary Information:**

The online version contains supplementary material available at 10.1007/s43615-021-00128-8.

## Introduction

Ideas of circular and thus more sustainable economies are widely promoted in policy, industry, and academia worldwide [1–3]. In China, circular economy (CE) is presented as one strategy to tackle urgent problems with environmental degradation and natural resource availability [2], in the European Union, CE “will make a decisive contribution to achieving climate neutrality by 2050 and decoupling economic growth from resource use” [1, p.2], and the Organisation for Economic Co-operation and Development (OECD) expects CE to contribute to “reducing atmospheric emissions, increasing the share of renewable energy and recyclable resources, as well as reducing the use of raw materials, water, land and energy” [3, p.1].

In the dominant discourse, at least in the West [4], circular economy is an eco-modernist technocratic concept [5, 6] resting on the idea that the current linear “extract-produce-use-dump material and energy flow model of the modern economic system is unsustainable” [7, p.37]. CE concepts posit that these unsustainable flows can be curbed and redirected by keeping services and products in use for longer and by reusing materials in new products and services over time. This can make for strange bedfellows where CE brings together environmental interests and heavy industry under a common umbrella of increased material circularity [8].

While CE involves diverse definitions, tools, and strategies [7, 9], almost all approaches involve a “R-framework”. A set of measures designed to close and slow material cycles in the economy—including reuse, repair, and recycling—are typically arranged in an order of priority or hierarchy. These frameworks are often abbreviated as xR (where x is the current number of strategies that an actor promotes in their definition of CE). The 10R framework for example covers refuse, rethink, reduce, reuse, repair, refurbish, remanufacture, repurpose, recycle, and recover. Despite the consistent inclusion of repair in such frameworks, policy, industry, and academia rarely focus on either the opportunities or the challenges involved in repair as part of an economic or cultural strategy to achieve circularity [10, 11], and so far, notwithstanding that CE has emerged as a central topic at the highest political and economic level [1–3], and recent measures regarding “Rights to Repair”, no government has set policy targets for repair [12]. If CE is to transform the relationships between ecological systems and economic activities, its political dimensions need to be better understood [5].

With a focus on repair, in this paper, through analysis of discussions on circular economies with Swedish repair stakeholders, we explore ways in which the existing political economy, cultural values, and future imaginaries enable or preclude consideration of different circular economy policy and practices.

A focus on repair offers two particular benefits in an exploration of the political economy of circularity. First, in contrast to many aspects of imagined forms of CE, repair already actually exists in the world; and second, repair provides a microcosm of the contestation over the political economy of circularity. At one extreme, corporate provision of product-service systems is presumed to incentivise durability and repair [13], and at the other extreme, repair is understood in terms of personal attachment to products expressed in “care-full” individual maintenance and repair (rich in attachment and informed by ethics of care) [14]. In between these extremes, there are existing modes of repair provision relying on the state, small businesses, or not-for-profit organisations such as repair cafés. Repair in practice embodies both the technocratic and convivial modes of circular economy suggested by Genovese and Pansera [5].

Our choice of Sweden for this study is partly a matter of convenience, but also reflects the country’s self-proclaimed CE leadership [8, 15], and tax incentives for repair which halve the rate of value-added-tax on repair, and provide tax deductions for maintenance and repair of homes [16]. These measures reflect Sweden’s long-standing aspirations to support both ecological citizenship, an approach to promoting pro-environmental behaviour rooted in changing attitudes through education to spread cosmopolitan values and associated social duties [17, 18], and ecological modernisation, the greening of the industrial economy, harnessing consumer choice [19, 20], as part of a distinctively Swedish “economic model” in which a tripartite state-business-labour consensus supports a significant welfare state, yet with strong emphasis on enabling new and small business formation in an internationally open competitive economy [21]. Despite increased market liberalisation, the impacts of the financial crisis, and emerging social fragmentation common to European states, here illustrated by the rise of the far-right Sweden Democrats as a significant political force, this consensus has been largely sustained to the present day [20].

By focusing on repair in Sweden, this paper offers analysis and critique of specific material circular economy approaches as implemented in a specific geographical and politico-economic context, alongside examination of expectations for future development rooted in an actually existing political economy. We examine the politics of repair as part of CE in Sweden using deliberative stakeholder workshops, which discussed distinctive future scenarios differing in the extent of centralisation or decentralisation of governance and the extent to which the political economy is sustained or transformed. The stakeholders involved included volunteer and professional repairers and craftsmen, repair scholars, and representatives of governmental agencies, environmental organisations, and companies with CE goals.

These discussions exposed tensions and challenges, as well as casting light on the social imaginaries and theories of change shared and presumed by our participants. These simultaneously reveal the significance of the situated context (in terms of the ideologies, interests, and institutions examined) and cast significant doubt on the effectiveness of Swedish efforts to promote ecological modernisation and ecological citizenship. Nonetheless, the values and ideals embodied by participants indicate that promotion of repair (in forms that directly engage individuals) might have valuable politically subversive effects, increasing support for a more interventionist state, and greater recognition for mending skills and manual labour involved.

In the following section, we present an overview of previous research on repair and circular economy and how this informs our theoretical understanding. Then, in the “Methodology” section, we present our methodology, including the scenarios and a presentation of how workshops were conducted. Results are detailed in the “Results” section, with a focus on the interplay of interests, institutions, and ideologies in establishing expectations of repair. In the “Concluding Discussion” section, we discuss the results with a focus on the future political economy of repair and CE and draw some conclusions.

## Theory: Previous Research on the Political Economy of Repair and Circular Economies

The mainstream academic literature on circularity and repair is remarkably technocratic and largely devoid of political analysis [10]. Circularity and, within that, an instrumental form of repair are presented as opportunities for a “win–win” outcome, in which economic and sustainability gains run hand in hand [7]. The politics of such a convergence are rarely considered. An interesting exception is Ghisellini et al.’s study which argues for an incorporation of circular economy in Keynesian expansionary fiscal policies in order to achieve a “sustainable welfare state” [22, p. 154]. Ghisellini and colleagues highlight that this would entail more active roles and shared responsibilities between consumers, industry, and political institutions. Nonetheless, there is an emerging literature which critically examines aspects of the political economy of circularity [5, 23, 24]. This section reviews existing work that exposes and explores dimensions of the contested political economy embedded in concepts of circularity and repair, to establish conceptual foundations for the empirical analysis which follows.

There are several key political and economic issues of relevance for CE: such as power relations between producers and consumers [25] and between sectors across value chains globally [26]; employment and labour issues [27, 28] including those in extractive industries, as well as in waste management and the informal sector [29]; and the management of environment and resources [30, 31]. These issues structure political interventions around circularity both directly and indirectly through the redistribution or consolidation of political power and influence. Despite the great relevance of such issues, studies directly addressing the political economy of CE are limited [5, 10, 23, 32]. This is a product both of the detachment of the CE concept from contested social, historical, or political relations and of a corporate focus on CE as an issue of innovation, business models, and product designs [10, 11]. Contestation in the CE space is reduced to the mere administrative management of effective markets in line with the advice of leading businesses, leaving little room for contestation in the political economy of neoliberal capitalism.

Previous research has recognised this depoliticised and technocratic character of CE [5, 8, 33]. Valenzuela and Böhm [23] interpret CE as a de-politicising strategy which re-organises and legitimises the continuation of an unsustainable capitalism, however under the guise of a “political economy of sustainability”. Genovese and Pansera [5] highlight that the dominant technocratic and eco-modernist representation of CE is not entirely hegemonic, with a state-directed “industrial ecology” framing significant in China’s state capitalist economy. Other scholars highlight the potential for contestation over the political economy of CE arising in community-based expressions of circularity or discourses of sufficiency and degrowth [e.g. 30]. This implies less focus on “how we produce” and more on “why or what we produce” [5, p.13]. CE can on the one hand “obfuscate…the continuity of capitalist interests”, but on the other hand, “‘circular’ values can be harnessed by local inhabitants to support their efforts” and lead to a “a more ‘embedded’ and diverse urban economy” [34, p.154]. The shapes of CE and repair depend on how they are embedded in political and economic structures.

For example, in recent years, circularity and repair have been promoted in Europe as a means to create jobs in a formal “green” economy, rather than as activities that might redistribute power to people as citizens acting outside the formal economy. In practice, the circular economy jobs that have been created so far are largely low-paid and low-skilled and mainly in the waste collection sector [35]. Similarly, in the CE literature, repair and circularity are most often understood in ways that reinforce consumer subjectivity, for example, emphasising consumer product life extension through repair and thus adding economic value [10]. Most CE repair literature and policies are focused on consumer goods and business models, emphasising market solutions for repair such as products-as-a-service systems [36, 37], “empowerment” of consumers through “right-to-repair” regulations [2, 38, 39], or standards for repairability labelling of products [40]. More transformative possibilities for repair are addressed by some scholars, examining the ways in which breakdown and repair of objects and infrastructures offer opportunities to reconsider economic and political systems and reconfigure cultural norms and behaviours [41–43].

In contrast to the instrumental discourses found in mainstream circular economies literature, sociological research on repair makes visible diverse and contested narratives and framings, in which repair is deeply embedded in relations with material artefacts, and social and political systems [44–46]. We have previously explored this theoretical “gap” between how repair is conceptualised in mainstream CE literature and policies on the one hand and how it is understood and presented in the emerging social research on repair on the other [10]. That research suggests that “repairers highlight the political opportunities that come in moments of breakdown – particularly to decide what should be restored, and what should be transformed”, and that repair could “…be a mechanism that responds to breakdown by enabling re-evaluation of how we interact in, and with this world” [11, p.10]. Repair is varied: it can be a sustaining or transforming process, it can be backward- or forward-looking, and it can have both a personal and political emphasis [10]. This indicates different roles for repair within the political economy: either sustaining, consumerist and nostalgic, or transformative, and future-oriented. Repair can thus be an important political change agent, e.g. as a sustainable alternative to use and dispose practices [47] and as a collective practice of community building and restoration [12]. To understand how repair can lead to a more circular economy in the future requires a better understanding of how it is embedded in political and economic structures beyond current framings of CE as a business model.

We draw on the studies presented in this section in order to analyse how relevant stakeholders understand the role of repair in the future and to discuss the political economy of repair. We consider both how existing ideologies, interests, and institutional structures condition these expectations and opportunities and how these could evolve or be reconfigured.

## Methodology

This study primarily utilised scenario workshops. A scenario is here understood as “a small bespoke set of structured conceptual systems of equally plausible future contexts, often presented as narrative descriptions, manufactured for someone and for a purpose…” [48, p.71]. For this paper, we constructed four “circular economy in the year 2050” scenarios (see descriptions below in the “The Circular Economy Scenarios: Repair in 2050” section). The scenarios enabled us to explore how different value sets and future imaginaries—especially with respect to the role and nature of repair—might generate different circular economy practices. To fulfil this purpose, we discussed the scenarios together with repair stakeholders at two workshops (for a description of the workshop method, see the “The Workshops” section).

### The Circular Economy Scenarios: Repair in 2050

**Fig. 1:**
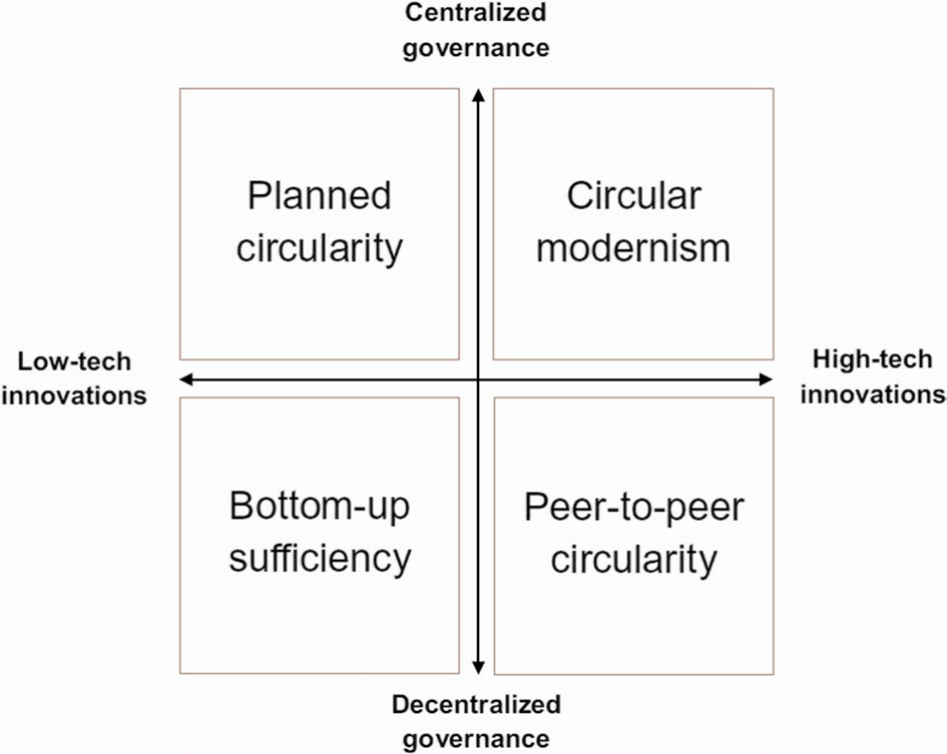
Bauwens et al.’s fourfold typology of circular economy scenarios (figure is from Bauwens et al. [49, p.6)

The scenarios are loosely based on Bauwens et al.’s [49, p.1] two-by-two scenario matrix (see Fig. 1) in which key dimensions of variation “…are the nature of technologies deployed – high-tech or low-tech innovations – and the configuration of the governance regime – centralized or decentralized”. Bauwens and colleagues developed four non-sector specific CE scenarios, “planned circularity”, “bottom-up sufficiency”, “circular modernism”, and “peer-to-peer circularity”, and tested them in focus group sessions involving CE experts, to ensure the plausibility of each scenario.

**Fig. 2:**
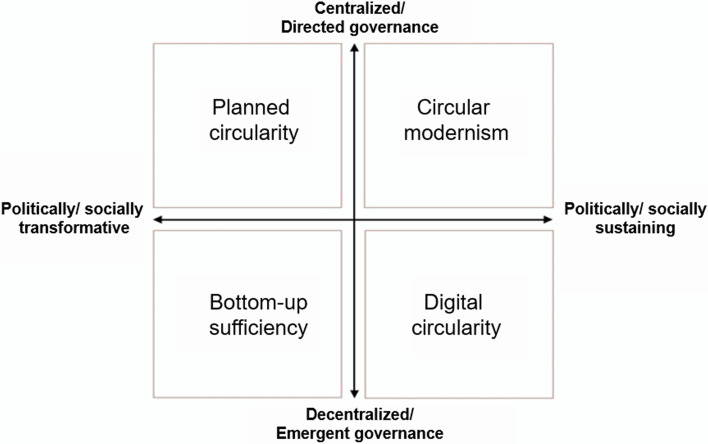
Fourfold typology of circular economy and repair scenarios

When designing our scenarios, we configured Bauwens et al.’s [49] four general scenarios to focus more specifically on repair (drawing on a set of interviews conducted earlier in our research, and reported in [11]) and to reflect the Swedish context, drawing on Swedish CE scenarios developed by Gunnarsson-Östling et al. [50] and Hagbert et al. [51]. On the innovation axis, rather than focusing on the low- or high-tech form of technology innovation, a rather arbitrary distinction, we highlight the politics of repair “innovations” as socially and politically *sustaining* or *transformative* forces. Although contemporary efforts to sustain political regimes in the face of environmental crisis often adopt visions of high-tech innovation as a tool [52], low-tech approaches can also form part of invocations of tradition as a conservative force. Nonetheless, a focus on repair as a socially disruptive or transformative force implies a different orientation to innovation at systemic scales, especially in terms of who participates and how it is funded, than a focus on sustaining existing structures and products [11, 53, 54]. We also wanted our scenarios to be less restricted in respect of the location of governance: we renamed Bauwens et al.’s [49] “peer-to-peer circularity” as “digital circularity” and refocused the scenario in order to recognise the power of digital platforms and to allow for diverse actors competing over political and economic influence, as seen in contemporary expressions of digital economies and technologies [55]. Our scenarios (presented in Fig. 2) are therefore distinguished on the axes of “centralised, or directed vs emergent or decentralised” and “politically/socially sustaining vs politically/socially transformative”.

The scenarios are not intended to be extreme “caricatures”, but to represent plausible futures characterised by possible developments on these dimensions [56]. As a result, each scenario contains its own tensions and potential contradictions (see Table 1 for detailed descriptions of the scenarios).

**Table.1:** Four futures of repair in the circular economy with key scenario characteristics

Circular modernism	Planned circularity	Bottom-up sufficiency	Digital circularity
• Everyday life in 2050 would seem similar in many respects to someone used to the present day	• Repair goals are embedded in policies and industries with a centralised “National Repair Service” ensuring availability of repair for infrastructures, buildings, and domestic products. Do-it-yourself repair has become less common	• Planned degrowth, voluntary simplicity, and minimalism have become dominant economic and cultural trends, as a response to long-term financial and economic crises	• Everyday life is based on digital platforms in this “app economy”. The platforms dominate a dynamic and diverse economy, in which people often consume online based services rather than owning products
• Repairability has become a significant element of “green” consumption, but durable, repairable products remain more prevalent in “high-end” markets, while mass consumption goods are designed for material recycling	• Everyday life has been transformed by increased public spending to support full employment, with a focus on supporting environmental goals. Many new state-supported jobs are created in environmental restoration and repair services	• Everyday life would seem very different from today. Most people live in more localised communities where repair as a necessity is part of everyday experience.	• Peer-to-peer sharing platforms and digital product leasing and service solutions are common. Digital platforms also enable trade in secondary materials and in repair work/services
• Repair as a hobby and professional repair coexist, but circularity is mostly delivered through recycling and reuse of materials, rather than repair and reuse of products	• The state intervenes more heavily in economic ownership: some businesses have been nationalised (and some closed) as part of a Green New Deal to repair common infrastructure	• Repair and maintenance of homes, material possessions, and local environment are core community values and objectives, and members of the community learn the skills they need to contribute	• Durability, repair, and material reuse are key strategic drivers for remaining manufacturers as the lifespan of each unit in circulation determines the profit margin. Many other products can be 3D printed, with circularity supported by recycling, rather than repair
• The economy is mixed: markets dominate resource allocation, but governments shape circularity through trade rules, product standards, and taxes	• Circularity, product durability, and design for repair are governed top-down through large scale, highly coordinated industry and municipal repair, recycling, and re-use programmes	• For most day-to-day needs, technology is small scale, affordable by locals, decentralised, labour-intensive, energy-efficient, and environmentally sound (such as bicycles and smaller wind turbines). Technology is often based on recycled materials, easy to maintain and repair	• Market-driven innovation, digitalisation, and automation in global markets have facilitated deployment of self-repairing materials and highly automated repair services
• Consumers are “nudged” through taxes and product labelling to “improve” consumption choices	• In sectors such as transport, collective solutions are centralised with fewer private vehicles, and consumption goods sectors are supported by mandatory take-back and repair systems	• Degrowth means that people have reduced consumption greatly. This drives do-it-yourself solutions and self-organisation. Wellbeing is measured not by consumption, but by care of—and meaningful participation in—ones community. Care for the elderly and children, and education, is shared by all	• Data protection and data security are major issues spurring controversies around the political power of monopolistic companies
National and European policy institutions continue in mix of cooperation and conflict, with continuing disagreements over the legality of national tax breaks on repair and over manufacturers’ efforts to force consumers to use certified repair services	• Corporate lobbies push back against the level of state ownership, while tensions remain over access to public services and employment guarantees for disadvantaged groups such as refugee and immigrant populations	• Much power has been delegated to citizens in local communities, but tensions still sometimes erupt over restrictions on resource overuse and environmental impacts. Young people often complain about the lack of opportunities they enjoy in comparison to their parents	• Political power largely remains with corporations, working closely with national governments, although the diversity of digital platforms has enabled decentralised experiments in participation and governance, as well as diverse economic identities and subcultures
		• Private property remains, but sharing of things is common, with tool and equipment libraries particularly carefully managed and maintained in most communities	• Political tensions between maker/hacker peer-to-peer networks and centralised platform monopolists are growing

### The Workshops

In the spring of 2021, two workshops were conducted in English using Zoom online meeting software with 16 and 15 participants, respectively. The workshops had been originally planned as face-to-face events, and invitations had been directed to stakeholders active in Sweden prior to a decision to conduct them online for COVID safety reasons. Our invitations took account of the desirability of diversity when it came to professions and gender. A total of nine men and 22 women participated representing different stakeholder groups: industry and labour organisations (2 participants); repair and climate grassroots (5); environmental and social NGO’s (7); SME’s (6), municipalities, and municipal companies (6); and scholars (5). In an effort to ensure credibility and reliability in the study results, we aimed to involve a broad variety of stakeholders relevant to the development of circular economy and repair in Sweden.

In preparation for the workshops, the scenarios were presented through a number of bullet points (see Table 1), a “news report image” (made at breakyourownnews.com), and a quote from an imagined involved character (using images from generatedphotos.com). One week before the workshops, each participant was assigned to a specific scenario and given a chance to examine it in advance of the workshop (scenarios were provided by email as pdf-files).

The workshop discussions were facilitated by the authors and two other academic colleagues to allow for guided discussions. Each workshop ran for four hours and consisted of both full group discussions and small group discussions. The workshops followed a set schedule of small group and full group discussion on the provided CE and repair scenarios; full group discussion on repair; and small group and full group discussions on policy measures. Discussions focused on the opportunities and challenges related to repair based on the different future scenarios.

### Analysis of the Workshop Discussions

Discussions during the workshops were audio recorded, transcribed, and anonymised for analysis. Participants’ written comments and contributions were collected on a joint digital “white board” during the discussions (using mural.com) and were also analysed. In the analytical phase of the study, we sought to gain insights about the political economy of repair as revealed in the expectations and interactions of key stakeholders. The coding and analysis of the transcribed material was done in three steps [57], to identify themes and repeating patterns in the stakeholder discussions. The first step, “manifest coding”, identified direct responses to specific questions on particular themes:

- What is repair now and in the future? (e.g. what is, and should be, repaired, and by whom?)
- How should repair be supported in the future? (e.g. through consumer behaviour, state infrastructures, digital applications, corporate innovation?)
- Which actors should support repair in the future? (e.g. state, corporations, local communities?)
- What structures, institutions or solutions should support repair in the future? (e.g. institutional developments, large-scale take-back infrastructures, small-scale political or business solutions, digital platforms, policy changes?)

In the second step, “global coding”, general traits among the different themes were identified. In this second step, we identified three categories of particular relevance: interests (political and economic winners and losers); institutions (organisations and systems of rules that structure social interactions); and ideologies (values and normative principles).

In the third, more analytical step, we identified the stakeholders’ social imaginaries and theories of change by analysing our categories for patterns, contradictions, and commonalities. The categories helped us analyse how diverse expectations of repair might be incorporated into, or unsettle the economic, political, and socio-technical systems which structure and permeate society. They also helped reveal participants’ shared understandings of how the world works and how it might be changed—in other words, their social imaginaries [58, 59] and theories of change [60, 61]. We use the term “theory of change” to describe a common understanding, not merely in its technical application as an evaluation tool. By distinguishing social imaginaries and theories of change, we draw attention to the potential for different stakeholders to understand differently how change happens [62] and to the prospect that different “theories of change” could be a source of contestation between stakeholders and between the participants and the convenors of the deliberative dialogues.

This three-step process is iterative: e.g. during the global coding, new keywords might be identified which can lead to the identification of new patterns, etc. In the “Results” section, we clarify whether presented viewpoints are common or less common amongst the stakeholders. All materials from the workshops have been analysed in this study, and theoretical saturation (i.e. the point when sampling more data will not lead to any more information) [63] was deemed to be reached when no additional viewpoints were identifiable within the material. The paper presents the data identified within the boundaries established by the study—the results rely on the methodological prerequisites presented in this section and, in particular, on the diversity of participants in the workshops. This is both a limitation of the study and an opportunity for further studies with additional or specific actor groups on specific aspects of the political economy of repair and circular economy. In order to strengthen reliability, all steps were conducted by both authors independently before synthesising the results.

However, it should be noted that our methodology does not seek to reveal some pre-existing objective reality regarding the opinions of stakeholders, but recognises that deliberative processes are spaces within which not only the opinions, but also the subjectivities and identities of stakeholders are co-produced [64]. Our deliberative methodology enabled interaction around potentially controversial stimulus material (the scenarios presented above) in an effort to enable strategic and collective thinking, not just to reveal pre-existing opinions and perspectives. In the following sections of this paper, we present the results and relate these to relevant CE and repair literature.

## Results

In this section, we present the results of the deliberative discussions. First, we briefly outline the main reactions of our stakeholders to the scenarios and indicate how these reactions might reflect the current political economy of repair in Sweden. Then, we provide a broader analysis of the ideologies, interests, and institutions addressed and implicated in the stakeholder discussions of future expectations and opportunities for repair and circularity in Sweden.

### Reactions

Stakeholder reactions to the different scenarios were diverse and in some respects even conflicting. Here, we briefly highlight a number of common responses that exemplify what we heard in both workshops.

#### Planned Circularity

“I don’t believe in the government’s ability to do this in an efficient way. I think they can do some things, but I think that it’s better that the market solves more of it. I don’t think they’re skilled enough in the government to solve this” (Participant Comment No. 1, PC1^1^).

While several participants found welcome aspects in the Planned Circularity scenario, and many endorsed a case for government intervention to support circularity and repair, rather than expecting individual action, there were concerns about implied restrictions on individual choice and the extent of state intervention. Many participants expressed a marked preference for market-based interventions and concern about the capacities and effectiveness of public sector solutions.

#### Digital Circularity

"Platforms and technology will also give the opportunity to be more suitable for each individual… So we can take personalized offers and setups that are more suitable [for the individual]” (PC2).

There was more consistent enthusiasm for the smart products and digitally enabled sharing of the Digital Circularity scenario as matching consumer demands and individuality, with little or no concern expressed about privacy issues. Concerns were expressed about the potential for increased corporate power in this scenario, and participants were somewhat divided on the benefits of product-service systems, some seeing a loss of valuable attachment, but in general, the scenario was seen as more in tune with current trends economically and technologically.

#### Bottom-up Sufficiency

“I think many people are really comfortable with their lifestyles like it is now” (PC3) … “it would be very hard to make the transfer to this scenario … the great institutions, like the big companies and the banks … will make great resistance [to] this transition” (PC4).

For many participants, this scenario appeared personally attractive but politically and practically implausible—with particular concerns raised about resistance by powerful interests, the acceptability to ordinary people of changed norms and low levels of consumption, and the ability of the scenario to maintain physical and social infrastructures, including the welfare state, and thus the potential for growing social inequality.

#### Circular Modernism

“This scenario’s emphasis on recycling, for me, that is not the whole circular economy, and really misses a big part about the speed and the levels of consumption” (PC5) … “I think that would be the biggest opportunity for repairing… to make repair as fun as consumption” (PC6).

This scenario felt very familiar to participants as an extension of current economic and technological trends, but for our groups, it did not go far enough to deliver circularity and repair, with its focus on material cycling, rather than product repair and reuse, although some saw opportunities to market repair directly to consumers. There were few comments on the politics involved, but many more on the practical measures are included, with many descriptions of existing obstacles to repair (e.g. cost, convenience, skills) and scepticism about the scope and sustainability of current initiatives to promote repair—especially by corporations.

The reactions to the scenarios reflect contemporary political debate and ideology in Sweden. We can hear echoes of a shared commitment to the welfare state and its safety nets but also of the ideology of individualism, such that state interventions focus on correcting market failures though measures like tax breaks that reinforce consumer subjectivity. We also heard many assertions—in reactions to all the scenarios—through which our participants distinguished themselves from “ordinary people”. Participants spoke and acted as thought-leaders or sustainability innovators, with presumed superior knowledge and normatively better values than those they wished to encourage to adopt goals of circularity and sustainability and associated norms. For example: “It’s not normal to fix things. It’s more normal to buy new things … Yeah, a lot of people are not like us” (PC7). Participants also repeatedly stressed the need for substantial changes in education and training to develop and share the skills and norms necessary to mainstream repair. For example: “not everyone has the skills or the ability, and a lot of things that people used to know, young people don’t know those things anymore” (PC8).

There is an obvious tension between the attachments revealed here to individualism, consumerism and the contemporary political economy of a wealthy European state, and the values and aspirations for change. In the following sections, we explore these tensions further by examining the relevant ideologies, interests, and institutions revealed and discussed by our stakeholders.

### Ideologies

By ideologies, we mean the value and belief sets expressed by stakeholders. Unsurprisingly, given our recruitment of stakeholders with interests in repair and circularity, a widely shared basic assumption is that CE in general and repair more specifically are “about sustainability and environmental impact” (PC9) and that such measures are normatively desirable for their contributions to achieving climate and environmental goals*.* But sustainability is itself a much debated and contested concept [65]. The group discussions reveal typically “weak” conceptions of sustainability in forms compatible with market liberalism, consumer choice, and individualism. Some participants posed direct challenges to this form of capitalism. But most at least presumed it as an inevitable part of the context for circularity and in many cases appeared even to endorse central aspects of the political economy implied.

For instance, we heard many more references to a need for skilled, specialised craftspeople, than arguments for self-reliance. Key elements of the industrial political economy, specialisation, a (global) division of labour, individualism expressed in career choices, and most critically consumer subjectivity, are all implicitly endorsed here. While consumerism was sometimes described as a problem in itself arising in “the basic principle of capitalism… that companies need to sell a lot in order to survive” (PC10), more typically participants sought measures such as standards, labelling, and tax differentials that would enable consumer power and consumer choice to drive circularity. In a consumer society, repair has to become “as much fun” as consumption (PC6, PC11, PC17), and the costs and inconvenience of repair to the consumer need to be overcome. Choice for the consumer might increase obstacles to repair, with a proliferation of non-standardised goods, but stakeholders interpret this as a consequence of corporate interests, not a systemic product of consumerism. Consumer choice, within an essentially capitalist economy, remains the way individuals express their values.

A norm of consumption rather than mending is understood to permeate Swedish society to the extent that repair is considered outdated, as one stakeholder put it “the wear-and-tear society was introduced in the 1950s when my grandparents were young and this was such a relief to them… of course you shouldn’t have to mend your socks … that’s a poor people thing to do” (PC12). Many contributions present repair as outside the norm in society, and some refer disparagingly to the consumption ideal of “western society” where we “have too much money” and it is “easy to buy cheap stuff” (PC13). But more often—echoing the findings of Dalhammar et al. [66] that repair experts see consumer demand as the main driver for promoting repair in Sweden—our stakeholders typically reaffirm a consumer subjectivity, in which “companies will redirect … their way of working if the consumers are demanding repairability” (PC14).

Against this background, a common understanding in the discussion is that there is a lack of awareness of repair possibilities amongst consumers due to societal norms. Increased consumer awareness is thus considered essential. There is a need to “educate people about [repair]” (PC15). Increased awareness and improved consumer information are not presented as a panacea, but as ways to close the gap between sustainability ideals and actions. As participants noted, there are multiple obstacles to becoming this “sophisticated” consumer: “people don’t know what they can repair and where they can find a repairer” (PC16), repairing, or finding a repairer is time-consuming, and “a struggle and no fun” (PC17) in a culture of time scarcity. Yet the norms of consumer convenience and the commodification of time are tightly bound together in contemporary political economy, and this may contribute to the common perception that self-provisioning and repair are not universally plausible routes to a circular economy and that an emphasis on the mainstreaming of professional repair is more important.

In this context, and in line with ecological citizenship theory, participants also placed emphasis on the potential to educate people and include repair skills in school curricula, “… add a block every week for mending stuff” (PC11). However, some stakeholders highlighted further obstacles and particularly questioned the scale of business response, noting that in practice where producers or retailers have developed circular business offerings, these are minor parts of their overall business, with “a huge discrepancy between the very small scale of what [companies] try, and the very large scale of their normal business… [material circularity] in no way impacts their main business model” (PC18). And similarly, that in practice, neither product service systems nor tax breaks have yet delivered significant increases in repair activity.

While these outcomes might reflect a lack of consumer demand, they also highlight that the vision of capitalist business following consumer demand is somewhat idealised, with many business strategies rather seeking to create markets for the resources and products companies have [67]. Yet many of our stakeholders expressed resignation regarding the prospect of regulating corporations, or unsettling the dominant position market-based solutions have in the circular economy. For some, this is also expressed through a belief that technology and innovation can solve the current sustainability crisis and that regulating individual behaviour is wrong: “I don’t believe that you should force anyone to do something. I think that technology [presents] the opportunity to give consumers more of what they need” (PC19).

While both “empowerment” and “agency” are terms used by stakeholders to describe preferable alternatives to centralised corporate or governmental power, this almost without exception is elaborated in terms of “consumer purchase power”, “increased consumer awareness”, or a “right-to-repair”. Consequently, the ideological position taken by many stakeholders is that individual action as consumers is the main solution, supported by market measures in government policy. Individuals have to demand improved consumer information or product labelling so they can “vote with their wallet”, “because in the end, it’s the consumer that decides what to buy” (PC20). The strong identification with individuals as consumers is perhaps not surprising considering that most stakeholders discuss repair in relation to household goods and electronics and few relate repair to, e.g. the restoration of nature or the maintenance of large-scale public infrastructures. However, the extent to which consumer subjectivity is reified and endorsed here has deep consequences for the potential political economy of repair. In our workshops, we saw more “ecological consumerism” than “ecological citizenship” [18]. Even if some participants themselves expressed personal values in line with the latter, their model or theory of change was primarily located in the former.

Besides being discussed as an issue of consumer norms and sustainability ideals, repair today inevitably involves labour. Stakeholders described repair as involving undervalued yet skilled manual labour conducted either by struggling small-scale craftsmen in waning local sectors, or as low-paid jobs exported to “foreign countries”. This orientation to repair-as-labour reflects consumption norms as well as an overvaluation of, and emphasis on, non-manual work and academic rather than technical qualifications in countries like Sweden. “Hobby” repair was not typically presented as a form of labour subjectivity, with only a minority of participants treating repair in this way at all. This has serious implications for the role of organised labour as a political actor in debates over repair and the CE. We heard wide agreement that “we need people to become craftsmen and to repair things”, although in practice, “people want craftsmen, but they don’t want to be a craftsman” (PC21). To address this, “society needs to value such skills” (PC22), and “we need to separate [professional] repair from hobby repair” (PC23). Repairers should be recognised as “professionals that will be demanded in the future” so that “young people will see it as a desirable [career choice]” (PC24). The ideal is that of a society with knowledgeable consumers demanding repair, supported by a competent labour force in which repair skills are respected and valued. But the dominant expectation amongst our participants was one in which demand for repair from consumers is the driving force, not corporate supply, nor organised labour interests.

In this respect, while our stakeholders sought change in norms, ideals, and behaviours in wider society, the underlying ideology of market liberalism and associated consumer subjectivity were only rarely brought into focus. Indeed as we saw above, scenarios that suggested potential to transcend such ideological norms were largely seen as implausible. In the next section, we turn to the interests identified and discussed and ask what scope there may be in this arena to intervene to support circularity and repair.

### Interests

Political economy analysis sees the intersection of political and economic influence and interests as critical to understanding outcomes. Here, we focus on the interests involved in circularity and repair and the expectations of stakeholders as to which interests might gain or lose in different scenarios, and how, and their likely responses. The manufacturing and related retail industries are understood by many stakeholders to be central actors, whilst extractive industries seem somewhat overlooked. Small business, labour, and civil society interests are also considered relevant.

The interests of manufacturing and retail industries were discussed primarily in the context of obstacles to repair that arise in production and distribution systems optimised for the modern linear global economy in which supplying cheap replacements manufactured in low-cost locations overseas is more profitable than repairing defective products and that such profits are protected by various ways in which manufacturers design and construct products that are inconvenient to repair. Most retailers also benefit from these systems, as they need not hold stocks of spare parts, nor pay for the labour skills needed for repair. Yet, as we have seen, many stakeholders still believe that circularity can be beneficial for these corporate interests too.

This tension comes to the fore in rich discussions of product service systems. Many stakeholders also believe that product-as-a-service business solutions will lead to extended producer responsibility for materials and that this increased corporate ownership will be “a possibility of developing professional repair service chains” (PC25) to simplify the provision of repair and that such business models provide an incentive to make products last because “it’s better for the economy of the companies” (PC26). The general argument is that this is a win–win situation which can serve companies and consumers and lead to environmental benefits [68]. However, the beliefs that increased ownership in the retail sector is of wider public interest and that products-as-a-service leads to extended producer responsibility were both questioned in our workshops. “Honestly”, demanded one participant “I would like to see some proof of it sometimes. That if the company owns it, they’re actually interested in making the product last longer … today, to date, I have not seen any evidence of it” (PC27). The same stakeholder argued that companies appear to have little interest in reducing the rate of throughput in such business models, noting that “the average lifetime of [rental] electric scooters are [only] 26 days. But [this] is baked into the price [of the service]” (PC27). There are disagreements regarding whose interest such business models in the retail sector would serve.

Manufacturing industries have different concerns regarding repair, mainly to do with that “labour is more expensive than capital”, and producers have diminished the share of labour costs through “being very efficient in the use of materials” and through “more intensive use of capital” (PC28). Intensive use of capital implies the outsourcing of repair to low-paying countries, “because in Sweden for example”, according to one industry stakeholder, “it’s very expensive to [own a] business: It’s hard to fire people… and you have the work environment laws… the production line in Sweden have so many regulations”, so to make a profit “companies put their production or the repair [in low-paying countries]” with less regulations (PC29). Others agree that repair requires “cheap labour in order to be affordable” (PC30). One stakeholder calls this the “paradox of repair”, i.e. that the repair of a product is labour intensive and therefore costly, while new products are cheap as new production “relies more on automatized production processes” (PC28). This means that there are few economic incentives for producers to increase repair in their business models or increase the reparability of their products, while there are incentives to automate production. These are, however, the same globalised corporations that typically lobby in favour of trade liberalisation [69].

The stakeholders typically aspired to support safe and secure labour conditions at the global as well as a national level and some supported tax reforms to reverse the balance of labour and materials costs in the market. A “change maker could be an increase of the price of materials”, or state decisions to “[move] the cost from labour to materials” (P28). This discussion situates repair as a public and labour interest constrained by growing corporate control over production and natural resources, with the private sector having “more power than some governments have” (PC31). There were associated worries expressed about how society could decarbonise if *“*the political power rests with corporations” (PC32), but, remarkably, no mention of likely political responses of extractive industries to interventions in material flows. And the expressed solution was to improve oversight of “… the state of our products and the whole value chain…” (PC33), enabling consumer leverage to ensure that “human rights, environmental laws and such are respected” (PC34). Unsustainable global extraction of natural resources was seen by some as a systemic challenge demanding “some other way of measuring or valuing economic activity” (PC35) and potentially a “global system” of regulations (PC36) to address material use, product quality and durability, repair, and the reuse of materials (PC37). The political challenges of inter-governmental negotiations in the face of powerful multi-national corporate lobbying were not considered. Yet a corporate-led circular economy was deemed to be unsustainable, as “every factory try to make their own little circle… this makes for poorer quality products which are also harder to repair” (PC37).

Despite concerns about labour conditions and employment opportunities globally as well as in Sweden, there was almost no consideration of organised labour and the possible role of trade unions. This appears to contrast with the relative significance of organised labour in the Swedish economic and political model, although it may be an artefact of the paucity of union representatives in our groups. The presence of repair business operators—by contrast—was reflected in repeated comments on skills, training, certification, and status for repair workers, such as “it’s impossible to have one person in each … shop that can repair. It’s impossible to find that kind of skills today” (PC38). Despite a view that repair skills are more widely available in other countries, and amongst immigrants because of necessity (PC39, PC40), repair was argued to be a means of retaining jobs in Sweden: “We have robots for many things, but repair, at least until this day requires people to work here as well. It’s harder to reallocate repairs to China” (PC41). But little else was said about working conditions or other political interests of labour as an interest group in a circular economy.

In contrast to unions, civil society organisations were understood as relevant interests. Several stakeholders run repair cafés and workshops, are engaged in repair through NGOs, or work with sustainable and fair trade organisations from positions in local government. While they have different organisational interests in repair, these stakeholders share more of a bottom-up perspective on the topic and agree that “power [should be] delegated to citizens and local communities” (PC42). In this discourse, people are presented as having more agency as repairers themselves and the use of local resources are emphasised. Civil society stakeholders argue for more “sufficiency discussions” amongst circular economy proponents (PC5). Still, from this perspective, voluntary provision is seen as insufficient and unsustainable: local initiatives “maybe won’t add up on the whole to become sustainable” (PC43), or that this will not address “how much materials we’re really using” (PC5)*.* Thus, stakeholders argue that measures are needed to enable community access to a wide variety of commercial repair services.

While our stakeholders recognised important interests in the political economy of circularity and repair, notably manufacturers and commercial repair services, others, such as labour unions or extractive industries, went largely uncommented upon. Although ideas of ecological citizenship were reflected more strongly in these aspects of the workshops, the consumer subjectivity still dominated. Below, we discuss the imaginaries and proposals of stakeholders for institutional change to support circularity.

### Institutions

Understood as the formal or informal “rules of the game” that structure human behaviour [70], institutions include not only specific organisations and rule-setting bodies or systems, but also norms and cultural and economic arrangements that act to enable or constrain our activities. As we saw at the end of the preceding section, civil society organisations and activities are seen as important but insufficient. Two broad institutions stand out as critical in the stakeholder discussions: markets and education. The measures or interventions proposed or expected largely follow from the ideals of liberal consumer sovereignty reported in the “Reactions” section. While stakeholders suggested multiple institutional measures to support repair, the majority fall into the model of a state intervening more-or-less vigorously to correct market failures and support “appropriate” consumer behaviour.

Suggested market-correcting and consumer-oriented measures included policies ensuring repairable and durable products with transparent expected life-times; regulation of producer responsibility banning planned obsolescence and preventing waste; voluntary product quality standards and labelling to increase transparency of material origin; financial support for repair services and business models that support repair; consumer information campaigns; fair trade policies; stricter procurement rules for public sector purchasers to help stimulate markets; and green tax-switching policies and tax cuts on repair work. Many such institutional measures are suggested or already implemented at different political levels today in various countries [71].

Despite a generalised preference for market- and consumer-oriented state interventions, stakeholders typically acknowledged a case for centralised maintenance of “infrastructure such as railways, highways, postal systems, and the internet” (PC44)*.* There were also indications of sympathy for more vigorous or larger-scale interventionism—reflecting broader political trends in which expansionary economic policies have been revived as “green new deals”. The EU Green Deal, for example, is presented as an action plan for a “clean, circular economy” [72], in response to financial and climate crises, and is now also positioned as tackling the economic effects of the coronavirus pandemic. The EU Green Deal is however also basically an expression of market liberalism, pinpointing consumer choice, the “right to repair”, consumers playing “an active role in the ecological transition” through informed choices*,* and new business models based on renting and sharing goods and services [38, p.8].

In a similar way, the state is also typically understood as the key actor delivering education in forms that support repair and a circular economy. The education system is seen as critical to awareness of the underlying environmental problems that necessitate circularity, as well as to awareness of the opportunities of repair. As one stakeholder put it, “I think it’s extremely necessary to be educated that things actually can be repaired. Because that builds for a future understanding that if something is broken, the first reply is not to toss it, but rather to find somebody who can repair it” (PC15). Education is also central to the development of the skills that will support a market-based circular economy. Stakeholders pointed to an overly academic education system as something itself in need of repair and suggested obligatory “repair classes” in schools, vocational repair training programmes, and certification of craft skills training to enhance recognition and uptake. One stakeholder argued that craft education should be available up to “the same as the master level at the university, so you can work with your hands and get such a high education and the [manual] skills as well” (PC45). Other related proposals to provide skills education included military-style “national service” focused on repair and the introduction of public repair jobs programmes. Nonetheless, such proposals do little to challenge the presumption that education is centrally about preparing people for roles as workers and consumers and, in the contemporary world, therefore emphasising skills for the “knowledge economy”, rather than manual and craft capabilities.

Amongst the stakeholders in our workshops, the main challenge to such mainstream discourses and centralised institutions came in the form of support from some for more radical municipal or community governance to mainstream “bottom-up initiatives”. Despite expressing ideological sympathy for such approaches, many stakeholders expressed significant pragmatic concerns. Those stakeholders active in these types of initiatives noted their continued reliance on both personal commitment and dependence on financial support from local and national “sustainability programmes”. Moreover, they highlight that such semi-institutionalised initiatives typically obtain only short-term support and are seen as experiments that must establish broader public or commercial support to merit continuation. While experimentation at least in theory “can challenge the status quo and enable the exploration of governance innovations, technologies and services in a temporary space” [73, p.17], and despite the existence of positive examples of business model experimentation within the circular economy space [74], our stakeholders expressed substantial fatigue with regard to their experiences of taking part in such sustainability experiments. Still, these stakeholders believe that the upscaling of local initiatives can be achieved through measures within the current institutional structure (through policies, taxes, and subsidies, etc.) (PC46, PC47).

The market-oriented proposals prioritised in our workshops reflect the style of existing institutional support for repair in Swedish policies—focused mainly on “soft policies” directed towards, for example, reparability of household electronics and consumer information. Our stakeholders tend to express dissatisfaction with the extent or vigour of existing policies, not the underlying presumptions and ideologies. These policies reflect an emphasis on standards in neoliberal environmental governance, in which “voluntary quality assurance standards covering flows of waste and resources around the globe are increasingly central to markets and trade” [75, p.1256]. Despite some challenging voices, the institutional arrangements of markets and liberal education emphasised in our discussions would seem likely to reinforce inadequate and unsustainable corporate models of circularity and a global political economy in which extractivism, productivism, and consumerism continue largely unchecked.

## Concluding Discussion

While our stakeholders sought change in norms, ideals, and behaviours in wider society, the underlying ideology of market liberalism and associated consumer subjectivity were rarely challenged [22]. The key interests associated with such ideologies—global manufacturing corporations—were understood as powerful and subject only to reformist market interventions. As within the mainstream CE discourse [10], most focus was placed on how to support repair and circularity through technological and business innovation or changes in consumer behaviour in ways that would enable continued business profitability, sustained employment, and a liberal welfare state. In other words, the dominant narratives of our workshops strongly reflected the political economy of market liberalism in the forms espoused in the so-called Swedish model [21].

The stakeholder discussions reported here thus suggest that even well-informed and well-intentioned sustainability pioneers within a wealthy Northern society find it almost impossible to escape the dominant ideologies and imaginaries of neoliberalism. The workshops provided an opportunity to reflect on and reconsider local, national, and European economic strategies, policy options, and business models. Yet the discussions rarely challenged conventional approaches. In this concluding section, we discuss further the underexplored tension between the dominant political economy and the goals of circularity and sustainability and reflect on implications of the subaltern narratives and imaginaries of repair and circular economy that circulated at the edges of our workshop conversations.

As we saw in the preceding sections, the stakeholder discussions functioned at two levels: at a dominant, articulated, and almost hegemonic level stakeholders volubly assert that producers, consumers, and politicians all want to act more sustainably and that repair and increased material circularity is one important strategy to achieve this. Within this agreement, detailed discussions arise about specific forms of taxes, business models, product designs, repair projects, etc. and how they relate to the production of repairable goods and changed individual consumer behaviour. Administrative and technocratic measures such as consumer nudging, quality assurance standards, and enhanced waste policies are all suggested in this neoliberal governance imaginary of sustainability [75]. Taken to an extreme, this imaginary accepts that corporations are so powerful that the only way to increase repair and circularity is through voluntary quality assurance standards established in conjunction with business interests. With this comes an anthropocentric and even consumer-oriented conceptualisation of sustainability in which “the valuation of ecosystem services is centred on their impact on subjective human [consumer] preferences, rather than in terms of their impact in the circular process of biophysical and socio-economic reproduction” [76, p.32]. This view flattens political, geographical, and socio-economic differences, as well as any difference between “natural capital” or “manufactured capital”, and thus rejects (or ignores) any need for political governance of the global extraction and (re-)use of resources. For the future of repair, appropriate interventions rest on further development of current economic-political trends, with the additional inclusion of specific “CE elements” such as digital platforms or product-as-a-service solutions.

The theories of change expressed and implied in these narratives are focused on ideas of individual behaviour change responding to improved knowledge and awareness but also pay attention to issues of convenience and recognise the significance of peer examples. However, they tend to fail to acknowledge the underlying play of political and economic interests that reinforce consumerist habits and actively maintain barriers to changes in behaviours and values. Thus, they cannot be expected to “close the gap” [77] between sustainability aspirations and action. Participants’ calls for better education simultaneously exemplify the limitations and hint at the possibilities. It is incontrovertible that new knowledge and skills are desirable, yet the political economy of contemporary education does not deliver the sort of knowledge, skills, and habitus needed in a genuinely circular society. But in the discussions, the idea that the education system itself is broken opened—albeit briefly—the prospect of a deeper analysis in which participants talked not only about the content of education but also about how the system shapes social values.

This was one example of a second, subaltern, level of discussion. In contrast with the dominant social and change imaginaries in which political economy is almost entirely absent, at this second level, typically on the margins of discussion, or as afterthoughts, stakeholders offered challenges, notably concerning the production, distribution, and use of materials at a global scale and how these relate to the varying power of different interests, governance at multiple levels, and social and environmental justice. In this subaltern discourse, the complex global network of ecological, economic, and political structures is in dire need of change, and repair and circularity are imagined as strategies or activities that can play a part in a transformation to a more just and ecologically sustainable politico-economic system, beyond flat sustainability targets. In this social imaginary, repair is understood as embedded in larger socio-technical, economic, and political systems in which the design and governance of these systems constrain and condition whether, how, and why repair can take place. At various moments in the workshops, mechanisms through which the dominant political economy prevents and resists repair and sustainability became visible. In particular, stakeholders highlighted the economic drivers for offshoring production and externalising costs, particularly through the exploitation of cheap labour reserves in the global south (both for production and for managing wastes). Discussions of consumer convenience exposed the ways in which current economic models both promote labour specialisation and manufacture time scarcity, by commodifying time, thus driving consumerism and purchase of services as opposed to self-provisioning (and repair). And in concerns raised by participants regarding the “bottom-up sufficiency” scenario, the somewhat paradoxical dependence of the welfare state on a growing economy to continue to fund pensions, health, and education emerged briefly as a problem.

There is a contradiction between the flat understanding of the world—with its focus on what is applicable, functional, and useful for consumers and producers—and the expressed (though rarely specified) need to transform current socio-political structures. The same unresolved contradiction lies at the core of the mainstream narrative on CE: i.e. that economic gains can be secured and true sustainability can be achieved, without achieving any systemic political, social, and economic change [5, 8, 23, 33]. As such, the stakeholder discussion analysed in this paper represents a microcosm of neoliberal environmental governance. We can however speculate that a different international composition—especially involving stakeholders from the global South—might have generated different insights and potentially more deeply challenged the mainstream narratives. This suggests that when Sweden or the EU proclaims leadership on CE, we should be alert to the problematic understandings and implications that brings.

The group composition was one of the limitations of this study we wish to highlight before closing. The stakeholders in our groups are all active in northern Europe, and this likely constrained the representations of repair and understandings of its political and economic relations in this study [cf. 11]. Moreover, within the groups, there were too few individuals from each distinct stakeholder group to establish differentiations between them. More replication of the workshops would have both enriched our data set, and perhaps enabled some initial comparison of sub-groups. Future research could usefully replicate such workshops in different international settings and/or increase the sample size so as to explore the specific understandings in these distinctive sub-groups. In addition, we want to acknowledge that the stakeholders have had no chance to provide any feedback to the conclusions of this article. While this is common to the research process, further discussions with these stakeholders (perhaps reconvening the groups after a period for reflection) would provide a valuable opportunity to surface subaltern narratives and dig deeper into how real structural change might be achieved.

The side-lining of challenging, subaltern views in these workshop discussions would also seem to confirm how the mainstream CE discourse can act as a powerful empty signifier [8, 23], convening different actors with diverse expectations of future strategies under a common umbrella. Not only does this ambiguity defer possible conflicts, it also means that CE acts as a label for business strategies and policy interventions which do more to protect the functioning of a specific form of neoliberal capitalism than they do to deliver sustainability. While the stakeholder discussions involved narratives in which increased repair is understood to be beneficial for the environment, consumers, workers, and corporations, such a win–win situation is far from certain. Such claims, made by actors such as the European Commission, that “[A] shared vision of the circular economy *can only boost* ongoing efforts to modernise the EU industrial base to ensure its global competitive edge and preserve and restore the EU’s natural capital” [78, p.11, emphasis added] are exposed as rhetorical efforts to avoid examining the continuing conflicts between global market capitalism and sustainability. In this analysis, the tools and market-based mechanisms of circular modernism advocated by Sweden and the EU seem unlikely to deliver the systemic change anticipated, and the depoliticising effects of Panglossian CE policy claims distract from very real tensions and conflicts that will need to be managed.

To understand these tensions and conflicts better, future research should explore further the role of manufacturing industries in constructing the unsustainable political economy of market liberalism. In addition, we suggest, first, further research to examine the role of extractive industries and associated interests in shaping discourses of circularity, rather than continuing to focus on consumer practices and behaviours and, second, research and policy that focuses on actively reducing linearity. Otherwise, as with the case of renewable energy and fossil fuels, circular solutions risk being “an extra cherry” on top of unsustainable solutions instead of a real transformation. As emphasised in the workshop discussions, CE and repair are trumpeted by many companies, but their core business models have yet to change.

This paper contributes to a richer theoretical understanding of CE by challenging the mainstream technocratic and individualistic understanding of circularity with ideas prevalent in the “sociology of repair” literature. An understanding of repair as deeply embedded in relations with material artefacts and social and political systems can lead to more diverse and contested circular economy policies and strategies. Prescriptive general conclusions about the practical implications for circular industry, policy, and civil society are difficult to present based on the studied material. However, we do see some hope for repair policies and practices to act in subversive ways, creating new opportunities to challenge and reconfigure the dominant political economy within practices which superficially appear to endorse and support existing power relations [79]. The subaltern discourse in our discussions with repair stakeholders suggests that framing understandings of brokenness not just as an instrumental need for repair but as an opportunity to highlight the politics behind the material arrangements could generate debate and help shift values and policies, on issues such as state intervention and technical education. Moreover, the study contributes to a much-needed debate about what brokenness is, what is broken in contemporary society, and the roles that different circular strategies can play in the transformation of these sectors. Such a reframing of brokenness might also help expose that the broken state of dominant ideologies and institutions is a persistent condition of a neoliberal political economy, therefore highlighting the inability of the system to repair itself despite continued invocations of technical innovation and consumer awareness.

## Supplementary Information

Below is the link to the electronic supplementary material.

Supplementary file1 (DOCX 17 KB)

## Data Availability

Workshop transcripts cannot be made available, as workshops were conducted under conditions of non-attribution.

## Footnotes

1 For detailed citation references, see Appendix. In the main text, the quotes are numbered PC1–47, and in the Appendix, we detail the workshop and session for each quote.
