# Supplementary material for: The Political Economy of Circular Economies: Lessons from Future Repair Scenario Deliberations in Sweden
Source: Circ Econ Sustain. 2021 Nov 10:1–25. Online ahead of print. doi: 10.1007/s43615-021-00128-8 (PMC8580547; doi:10.1007/s43615-021-00128-8)
Supplement: Supplementary file 1 — Supplementary file1 (DOCX 17 KB) [file 43615_2021_128_MOESM1_ESM.docx]

# Appendix A – Empirical material: participant comments

**List of abbreviations**

BUS – Bottom-up Sufficiency scenario session

CM – Circular Modernism scenario session

DC – Digital Circularity scenario session

PC – Planned Circularity scenario session

J – Joint session with participants from all scenario sessions

**Table 1. Participant citations**

| Participant Comment No. | Workshop No. | Session |
| --- | --- | --- |
| 1 | 2 | PC |
| 2 | 1 | DC |
| 3 | 2 | BUS |
| 4 | 1 | BUS |
| 5 | 2 | CM |
| 6 | 1 | CM |
| 7 | 1 | BUS |
| 8 | 2 | PC |
| 9 | 1 | DC |
| 10 | 2 | CM |
| 11 | 1 | J |
| 12 | 1 | PC |
| 13 | 1 | J |
| 14 | 1 | BUS |
| 15 | 2 | J |
| 16 | 1 | CM |
| 17 | 2 | J |
| 18 | 1 | CM |
| 19 | 1 | DC |
| 20 | 1 | J |
| 21 | 1 | CM |
| 22 | 1 | PC |
| 23 | 1 | J |
| 24 | 1 | BUS |
| 25 | 1 | CM |
| 26 | 1 | CM |
| 27 | 1 | CM |
| 28 | 1 | J |
| 29 | 1 | DC |
| 30 | 1 | DC |
| 31 | 1 | DC |
| 32 | 1 | DC |
| 33 | 1 | DC |
| 34 | 1 | DC |
| 35 | 2 | CM |
| 36 | 2 | CM |
| 37 | 2 | CM |
| 38 | 1 | BUS |
| 39 | 2 | PC |
| 40 | 2 | J |
| 41 | 1 | J |
| 42 | 1 | BUS |
| 43 | 1 | BUS |
| 44 | 1 | BUS |
| 45 | 2 | J |
| 46 | 2 | J |
| 47 | 1 | PC |
